# Network analysis of lifestyle behaviors and anxiety in children and adolescents: gender and school-stage heterogeneity

**DOI:** 10.3389/fpsyg.2026.1803654

**Published:** 2026-05-18

**Authors:** Liping Yu, Jun Chen, Lulu Gan, Yue Xi

**Affiliations:** 1 School of Physical Education, Guangxi University, Nanning, China; 2 Postdoctoral Research Station of Physical Education, Fujian Normal University, Fuzhou, China; 3 School of Sport and Physical Education, Shanghai University, Shanghai, China

**Keywords:** anxiety, children and adolescents, lifestyle behaviors, network analysis, physical activity

## Abstract

**Background:**

Anxiety disorders are a growing public health concern among children and adolescents, and lifestyle behaviors are recognized as modifiable factors. However, how lifestyle behaviors and anxiety symptoms interrelate, and whether these associations differ by gender and developmental stage remains unclear.

**Objective:**

This study examined the network structure of lifestyle behaviors (physical activity, sleep, diet) and anxiety symptoms in children and adolescents, examining gender- and school-stage-specific differences.

**Methods:**

A total of 1,971 children and adolescents from China (50.8% male, 49.2% female; 69.8% primary school, 30.2% middle school) participated in this cross-sectional study. Lifestyle behaviors were assessed using the International Physical Activity Questionnaire-Short Form (IPAQ-SF) and self-developed composite indices for sleep (4-item) and diet (3-item) behaviors, while anxiety symptoms were measured using the Generalized Anxiety Disorder-7 scale (GAD-7). Network models were estimated using regularized partial correlations. Expected influence (EI) and bridge EI were used to identify central and bridge nodes.

**Results:**

Moderate-intensity physical activity (MPA) emerged as the most central node (EI = 1.10), followed by anxiety symptoms GAD06 (EI = 0.96) and GAD03 (EI = 0.89). Sleep was identified as the strongest bridge node (bridge EI = −0.230), showing the strongest cross-domain partial correlation between lifestyle behaviors and anxiety symptoms. Gender-specific analyses showed significantly stronger sleep bridge effects in females (bridge EI = −0.270) than in males (bridge EI = −0.190, *p* = 0.033), despite comparable overall network structures (*p* = 0.263). Developmental comparisons indicated that primary school students exhibited stronger behavior-symptom associations, whereas middle school students showed more consolidated symptom-symptom connectivity. All networks demonstrated good stability (CS-coefficients: 0.67–0.75).

**Conclusion:**

Sleep was the most prominent bridge node linking lifestyle behaviors and anxiety symptoms, with gender-specific and developmental differences observed. These findings are descriptive and hypothesis-generating; claims regarding mechanisms or intervention targets require longitudinal and experimental evidence.

## 1 Introduction

Over the past 5 years, mental health problems among children and adolescents have increased substantially, with anxiety disorders representing the most prevalent psychopathological condition in this population. A landmark meta-analysis published in JAMA Pediatrics reported that during the COVID-19 pandemic, the global prevalence of anxiety symptoms among children and adolescents doubled to 20.5%, indicating that one in every five youths experienced clinically significant anxiety (Racine et al., 2021). This increase is not a transient fluctuation but reflects a decade-long deterioration in youth mental health, particularly pronounced in high-income and middle-income countries (World Health Organization, 2022). From a public health perspective, this trend threatens progress toward UN Sustainable Development Goal 3.4. Anxiety contributes substantially to disability-adjusted life years and predicts suicidality, academic underachievement, and adult cardiovascular disease (Kalin, 2021; Karlsen et al., 2021; Pagerols et al., 2022). Yet fewer than 20% of affected children receive treatment (World Health Organization, 2022).

Traditional clinic-based intervention models—constrained by resource shortages and limited accessibility—are insufficient to meet the escalating mental health needs of youth. This has increased interest in scalable, low-cost strategies rooted in lifestyle-based prevention. Substantial evidence indicates that healthy lifestyle behaviors protect against anxiety and related emotional problems. Engagement in moderate-to-vigorous physical activity is consistently associated with lower anxiety and depression (Yang et al., 2025), whereas insufficient or irregular sleep predicts elevated anxiety symptoms (Yang et al., 2025). Diet also plays a critical role: studies among Chinese children show that low dietary diversity and frequent consumption of ultra-processed foods markedly increase the risk of mental health problems. Overall, unhealthy lifestyle patterns—including physical inactivity, sleep disturbances, and poor diet—are strongly linked to heightened anxiety in adolescents (Huang et al., 2025). However, three critical limitations characterize this literature. First, most studies have examined lifestyle behaviors in isolation, treating them as independent predictors rather than an interconnected system (Hoare et al., 2016). This approach overlooks how these behaviors mutually influence each other—for example, poor sleep may reduce physical activity, while dietary patterns affect both sleep and energy levels. Second, existing research has predominantly relied on sum scores to represent anxiety, obscuring symptom-specific associations (Fried and Nesse, 2015). Identifying which specific symptoms are most strongly linked to lifestyle behaviors could inform more targeted interventions. Third, while mean-level differences in anxiety across gender and developmental stages are well-documented, whether the structural relationships among lifestyle behaviors and anxiety symptoms differ across these groups remains unexplored. Such structural differences would have important implications for tailoring interventions to specific populations.

Gender and developmental stage may further shape these associations. Female adolescents report higher prevalence and severity of anxiety than males (McLean and Anderson, 2009), and the associations between physical activity and anxiety appear to differ by gender, with varying effect sizes between boys and girls (Carter et al., 2021). Similarly, sleep-anxiety associations differ by gender, with stronger relationships observed among adolescent females (Zhang et al., 2017). Developmentally, the relationship between sedentary behavior and mental health outcomes strengthens from childhood through adolescence (Biddle et al., 2019). Recent network studies have also demonstrated that symptom network structures differ by both age and gender (Garabiles et al., 2019; Mullarkey et al., 2019). These findings highlight the need to examine lifestyle-anxiety network structures separately by gender and developmental stage.

Traditional linear models assess only average effects of individual predictors, limiting their ability to capture complex interdependencies (Li et al., 2025). Network analysis treats lifestyle variables and anxiety symptoms as nodes, with edges representing direct associations. This approach calculates centrality and bridge indices that may help identify influential nodes and connections linking lifestyle and anxiety subnetworks (Yang et al., 2025). Network analysis has proven valuable in psychopathology research, successfully identifying bridge symptoms that connect physical activity with anxiety and depression (Kaiser et al., 2021; Yang et al., 2025). This study aimed to: (1) construct a comprehensive network integrating five lifestyle dimensions (vigorous, moderate, and light physical activity, sleep, and diet) with seven anxiety symptoms (GAD-7) to examine how these variables are interconnected as an integrated system; (2) systematically compare network structures across gender and school stage (primary vs. middle) using Network Comparison Tests, identifying development- and gender-specific patterns to identify candidate bridge nodes for future longitudinal and experimental investigation; (3) employ bridge centrality analysis to identify key bridge nodes connecting lifestyle and anxiety domains that may warrant further longitudinal and intervention research. These analyses were intended to move beyond documenting associations between lifestyle behaviors and anxiety, toward understanding patterns of interconnection and potential variation across subgroups.

## 2 Method

### 2.1 Participants and procedure

This cross-sectional study was conducted in August 2025 in a prefecture-level city in southwestern China. Using convenience sampling, we recruited participants from both urban and rural primary schools (grades 1–6) and middle schools (grades 7–9). The inclusion criteria were: (1) enrolled students in grades 1–9; (2) voluntary participation with informed consent. Exclusion criteria included: (1) diagnosed psychiatric disorders requiring clinical treatment; (2) severe cognitive impairment preventing questionnaire completion; and (3) incomplete survey responses (>20% missing data). Of 2,156 initial respondents, 185 were excluded due to incomplete or inconsistent data (e.g., missing items or uniform response patterns across all items), yielding a final sample of 1,971 students (response rate: 91.4%). The final analytic sample contained no missing data on any study variables. Sample characteristics are shown in Table 1. Participants' mean age was 10.42 ± 2.08 years. The sample comprised 1,002 males (50.8%) and 969 females (49.2%). By ethnicity, 59.0% were Han Chinese, 15.53% were Zhuang, and 25.47% were from other ethnic groups. Most (75.19%) came from multi-child families. By school stage, 1,375 (69.8%) were primary school students (grades 1–6) and 596 (30.2%) were middle school students (grades 7–9). Within the primary school sample, 289 students (14.7% of total) were in grades 1–2. Questionnaires were administered collectively in classrooms during school hours under trained staff supervision. For students in grades 1–2, pinyin annotations were provided to facilitate comprehension. Teachers were instructed to read items verbatim without providing interpretations or guiding responses. Questionnaires were completed under standardized classroom conditions, with age-appropriate assistance available when necessary. This study involving human participants was reviewed and approved by the Ethics Committee of Guangxi University (Approval No.: GXU-2025-088) and adhered to the Declaration of Helsinki. Written parental consent and student assent were obtained prior to participation.

**Table 1:** Descriptive characteristics of studied variables.

Characteristics	*N* = 1971	Node	Abbreviation	Total	Male	Female	p*(d)*	Primary	Middle	*p (d)*
	M ±SD/n(%)			*N* = 1971	*N* = 1002	*N* = 969		*N* = 1375	*N* = 596	
				M ±SD				M ±SD		
Demographic information		lifestyle								
Age (years)	10.42 ± 2.08	vigorous-intensity physical activity	VPA	581.87 ± 1339.51	658.81 ± 1452.01	502.32 ± 1207.87	0.009(0.117)	538.59 ± 1382.10	681.73 ± 1230.99	0.029(−0.107)
Race		Moderate-intensity physical activity	MPA	288.44 ± 646.15	293.15 ± 539.29	283.58 ± 740.87	0.742(0.015)	253.01 ± 651.01	370.18 ± 627.75	0.001(−0.182)
Han	1163(59%)	Light-intensity physical activity	LPA	270.14 ± 606.37	283.60 ± 604.77	256.22 ± 608.01	0.316(0.045)	234.63 ± 532.04	352.06 ± 744.35	0.001(−0.194)
Zhuang	502(15.53%)	Sleep behavior	SLEEP	7.91 ± 1.37	7.94 ± 1.34	7.88 ± 1.40	0.349(0.042)	7.93 ± 1.38	7.86 ± 1.86	0.328(0.048)
Other	306(25.47%)	Dietary behavior	DIET	9.20 ± 2.09	9.16 ± 2.01	9.24 ± 2.11	0.415(−0.037)	9.14 ± 2.04	9.35 ± 2.19	0.046(−0.101)
Number of children		*GAD-7*								
Only child	489(24.81%)	GAD01-anxiety	GAD01	1.42 ± 0.78	1.39 ± 0.74	1.46 ± 0.81	0.026(−0.101)	1.39 ± 0.76	1.49 ± 0.83	0.013(−0.127)
Multiple children	1482(75.19%)	GAD02-uncontrollable worry	GAD02	1.34 ± 0.72	1.33 ± 0.71	1.35 ± 0.73	0.466(−0.033)	1.31 ± 0.69	1.41 ± 0.79	0.010(−0.134)
		GAD03-generalized worry	GAD03	1.42 ± 0.76	1.40 ± 0.75	1.44 ± 0.78	0.177(−0.061)	1.39 ± 0.73	1.50 ± 0.84	0.005(−0.146)
		GAD04-trouble relaxing	GAD04	1.39 ± 0.76	1.37 ± 0.73	1.42 ± 0.79	0.125(−0.069)	1.35 ± 0.71	1.49 ± 0.87	0.001(−0.196)
		GAD05-restlessness	GAD05	1.33 ± 0.72	1.34 ± 0.74	1.33 ± 0.71	0.803(0.011)	1.32 ± 0.70	1.36 ± 0.77	0.280(−0.053)
		GAD06-irritability	GAD06	1.45 ± 0.82	1.43 ± 0.80	1.48 ± 0.84	0.124(−0.069)	1.42 ± 0.79	1.53 ± 0.88	0.014(−0.127)
		GAD07-fear of awful events	GAD07	1.31 ± 0.71	1.31 ± 0.69	1.31 ± 0.72	0.984(−0.001)	1.29 ± 0.67	1.37 ± 0.79	0.028(−0.115)

### 2.2 Measurement

Physical activity was assessed using the International Physical Activity Questionnaire-Short Form (IPAQ-SF) (Craig et al., 2003), a widely used instrument that has been validated in Chinese populations including adolescents (Macfarlane et al., 2007). The IPAQ-SF consists of seven items assessing physical activity over the past seven days. The questionnaire records the frequency (days/week) and duration (minutes/day) of three activity intensities: (1) vigorous-intensity activities (VPA), defined as activities that make you breathe much harder than normal, such as running, fast cycling, aerobics, or competitive sports; (2) moderate-intensity activities (MPA), defined as activities that make you breathe somewhat harder than normal, such as carrying light loads, cycling at a regular pace, or recreational swimming; and (3) light- intensity physical activity, including walking at home, walking to travel from place to place, and any other walking for recreation or exercise. According to the IPAQ scoring protocol, standard Metabolic Equivalent (MET) values were assigned as follows: 3.3 METs for walking, 4.0 METs for moderate activity, and 8.0 METs for vigorous activity. Weekly physical activity (MET-min/week) was calculated using the formula: MET value × days per week × minutes per day, following the official IPAQ guidelines and the calculation principles proposed by Fan et al. (2014). Higher MET-min/week values indicate a greater volume of physical activity.

Lifestyle behaviors, including healthy sleep behaviors and healthy eating habits, were assessed using brief self-developed items designed for use in a school-based survey of Chinese children and adolescents. These variables should be interpreted as pragmatic composite indicators rather than comprehensive validated measures of broader behavioral constructs. Sleep behaviors were assessed using four items measuring distinct dimensions of sleep: (1) daily sleep duration including naps (“≥8 h” = 1, “ <8 h” = 0); (2) sleep environment, specifically sleeping with lights on (no = 1, yes = 0); (3) regularity of sleep schedule (1 = never to, 4 = always); and (4) frequency of insomnia or poor sleep quality (reverse-scored: 4 = never to, 1 = always). A composite score was calculated by summing all items (range: 0–10), with higher scores indicating healthier sleep behaviors. The scale demonstrated acceptable internal consistency in the present sample (Cronbach's α = 0.713). Diet behaviors were assessed using three items: (1) “How often do you have three meals at regular times each day?”; (2) “How often do you pay attention to nutritional balance in your diet?”; and (3) “How often do you eat vegetables and fruits?”. Each item was rated on a 4-point frequency scale (1 = never, 2 = occasionally, 3 = often, 4 = always). A composite score was calculated by summing all items (range: 3–12), with higher scores indicating healthier dietary behaviors. The scale demonstrated good internal consistency in the present sample (Cronbach's α = 0.813). Inter-item correlations for the sleep scale ranged from 0.21 to 0.52, whereas those for the diet scale ranged from 0.53 to 0.63, indicating greater heterogeneity for sleep and relatively stronger coherence for diet. Correlations between the sleep and diet composite scores and the GAD-7 total score were in the expected negative direction (*r* = −0.34, *p* < 0.001; *r* = −0.19, *p* < 0.001, respectively), providing preliminary evidence that these indicators were related to anxiety symptoms in theoretically expected ways. Internal consistency across school stages was relatively stronger for diet than for sleep (primary/middle school: diet α = 0.817/0.801; sleep α = 0.721/0.658).

The GAD-7 was originally developed as a brief self-report measure of generalized anxiety symptoms in adults (Spitzer et al., 2006) and has since shown acceptable psychometric performance in adolescent and youth samples (Mossman et al., 2017; Sun et al., 2021). Evidence from Chinese youth and adolescent samples has also supported its reliability and validity (Sun et al., 2021; Ip et al., 2022). However, direct validation studies in children younger than 10 years remain limited. Therefore, in the present sample, we further examined its performance by reporting internal consistency across age/grade groups. The GAD-7 showed excellent internal consistency overall (Cronbach's α = 0.912), with corrected item-total correlations ranging from 0.70 to 0.76. Reliability was also good across the three age/grade subgroups (α = 0.918, 0.905, and 0.927, respectively). Although these findings provide additional support for use of the GAD-7 as a brief symptom measure in the present sample, formal measurement invariance testing was not conducted, and results for the youngest participants should still be interpreted with caution.

### 2.3 Data analysis

#### 2.3.1 Network estimation

Missing data were handled using list-wise deletion. The Anderson–Darling test indicated that all variables significantly deviated from normality (*p* < 0.05); therefore, Spearman correlations were used to estimate associations among lifestyle behaviors (diet, sleep, physical activity) and anxiety symptoms. Networks were constructed from regularized partial correlation matrices using the graphical LASSO and visualized with the qgraph package in R (Epskamp et al., 2012). A single integrated network was estimated with nodes representing lifestyle behavior indicators (VPA, MPA, LPA, sleep, and diet) and GAD-7 anxiety symptoms. Nodes represented lifestyle indicators and GAD-7 symptoms, and edges represented regularized partial correlations controlling for all other variables. Regularization was applied to improve sparsity and minimize multicollinearity (Epskamp and Fried, 2018).

#### 2.3.2 Network centrality

We computed strength and expected influence (EI) to assess node importance. EI was used as the primary centrality index because it preserves the sign of edge weights and is therefore more suitable for networks containing both positive and negative associations (Hevey, 2018). Strength centrality, in contrast, reflects the absolute sum of edge weights. We further calculated bridge expected influence to quantify the extent to which nodes in one community influence nodes in another. In this study, bridge EI was calculated to quantify the extent to which nodes in the lifestyle behavior domain (VPA, MPA, LPA, sleep, diet) influence nodes in the anxiety symptom domain (GAD01–GAD07), and vice versa. Communities were defined a priori based on the theoretical distinction between lifestyle behaviors and psychological symptoms. All analyses were performed using the R package “qgraph” (version 1.9.8) and “bootnet” for network estimation and stability assessment.

#### 2.3.3 Network comparison and sensitivity analyses

We employed the Network Comparison Test (NCT) to examine potential differences in network structure, global strength, and edge strength across gender and school-stage groups (van Borkulo et al., 2014). The NCT used a permutation-based approach with 1,000 iterations. The Benjamini–Hochberg false discovery rate (BH-FDR) correction was applied to adjust for multiple testing in the edge-wise comparisons. Additional sensitivity analyses included: (a) excluding grades 1–2 and re-estimating the network; (b) inspecting item-level GAD-7 response distributions across school-stage subgroups; (c) entering the constituent sleep- and diet-related items separately into the network; and (d) conducting a stage-adjusted sensitivity analysis in which each node was residualized for stage and the network was re-estimated using the residualized variables.

#### 2.3.4 Network accuracy and stability analyses

The correlation stability coefficients (CS-coefficient) for centrality and bridge centrality indices were computed to estimate network accuracy, with a value above 0.25 as acceptable and above 0.50 as preferable (Epskamp et al., 2018). We also calculated bootstrapped confidence intervals (CIs) of network edge-weights to examine network accuracy (Epskamp et al., 2018).

## 3 Results

### 3.1 Sample characteristics

Table 1 presents the descriptive characteristics of the study variables. The total sample consisted of 1,971 children and adolescents (50.8% male), including 1,375 primary school students (69.8%) and 596 middle school students (30.2%). Regarding lifestyle behaviors, males reported significantly higher levels of VPA than females (658.81 ± 1452.01 vs. 502.32 ± 1207.87 MET-min/week, *p* = 0.009), whereas no significant gender differences were observed for MPA, LPA, sleep, or dietary behaviors. When comparing school stages, middle school students reported significantly higher levels of VPA (*p* = 0.029), MPA (*p* = 0.001), LPA (*p* = 0.001), and healthier dietary behaviors (*p* = 0.046) relative to primary school students.

For anxiety symptoms, females showed significantly higher scores on GAD01 compared with males (1.46 ± 0.81 vs. 1.39 ± 0.74, *p* = 0.026). Middle school students reported significantly higher scores across most anxiety dimensions, including GAD01 (*p* = 0.013), GAD02 (*p* = 0.010), GAD03 (*p* = 0.005), GAD04 (*p* = 0.001), GAD06 (*p* = 0.014), and GAD07 (*p* = 0.028), indicating a developmental increase in anxiety severity. Overall, while several group differences reached statistical significance, all observed effect sizes were small to negligible, suggesting limited practical significance of these differences.

### 3.2 Overall network structure and central symptoms

**Figure 1:**
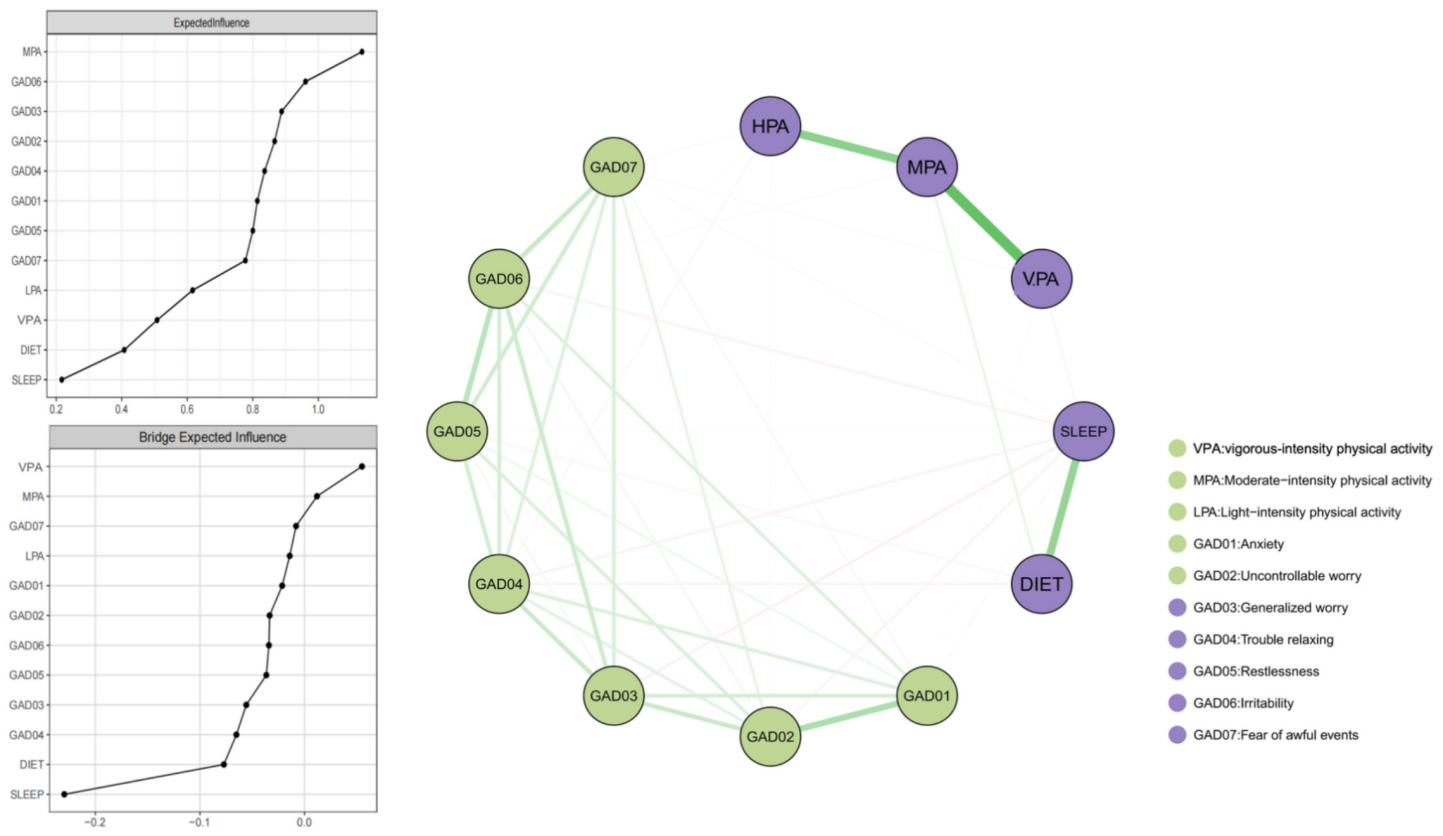
Network model and centrality indices (EI and bridge EI) for lifestyle behaviors and anxiety symptoms. Green edges represent positive associations while red edges represent negative associations. Thicker edges represent stronger association.

Figure 1 presents the overall network structure of lifestyle behaviors and anxiety symptoms. EI analysis identified MPA as the most central node (EI = 1.10). A cluster of anxiety symptoms—GAD06 (EI = 0.96), GAD03 (EI = 0.89), and GAD02 (EI = 0.87)—exhibited the strongest within-domain connectivity. In contrast, lifestyle behaviors (sleep, diet, VPA, LPA) had substantially lower EI values, reflecting weaker global influence (Supplementary Material 4). Bridge EI revealed that sleep served as the strongest bridge node (bridge EI = −0.230), exhibiting the strongest bridge expected influence value between lifestyle behaviors and anxiety symptoms. Diet (bridge EI = −0.077) also demonstrated a meaningful bridging effect, while among anxiety symptoms, GAD04 (bridge EI = −0.065) and GAD03 (bridge EI = −0.056) were the strongest bridge nodes within the anxiety domain. Physical activity variables showed minimal bridge influence (bridge EI <0.06). The strongest positive edges in the network were VPA-GAD04, VPA-GAD07, and MPA-GAD06, whereas the strongest negative edges were SLEEP-GAD03, SLEEP-GAD04, and DIET-GAD04 (Supplementary Material 2, 3). A supplementary item-level sensitivity analysis indicated that the constituent sleep- and diet-related items did not contribute uniformly, particularly for sleep. The corresponding item-level centrality and bridge results are presented in (Supplementary Material 16). In addition, in a stage-adjusted sensitivity analysis, the overall network pattern remained broadly similar. Sleep remained the strongest bridge node, and MPA remained the most central lifestyle-related node.

### 3.3 Network structure analysis across gender groups

**Figure 2:**
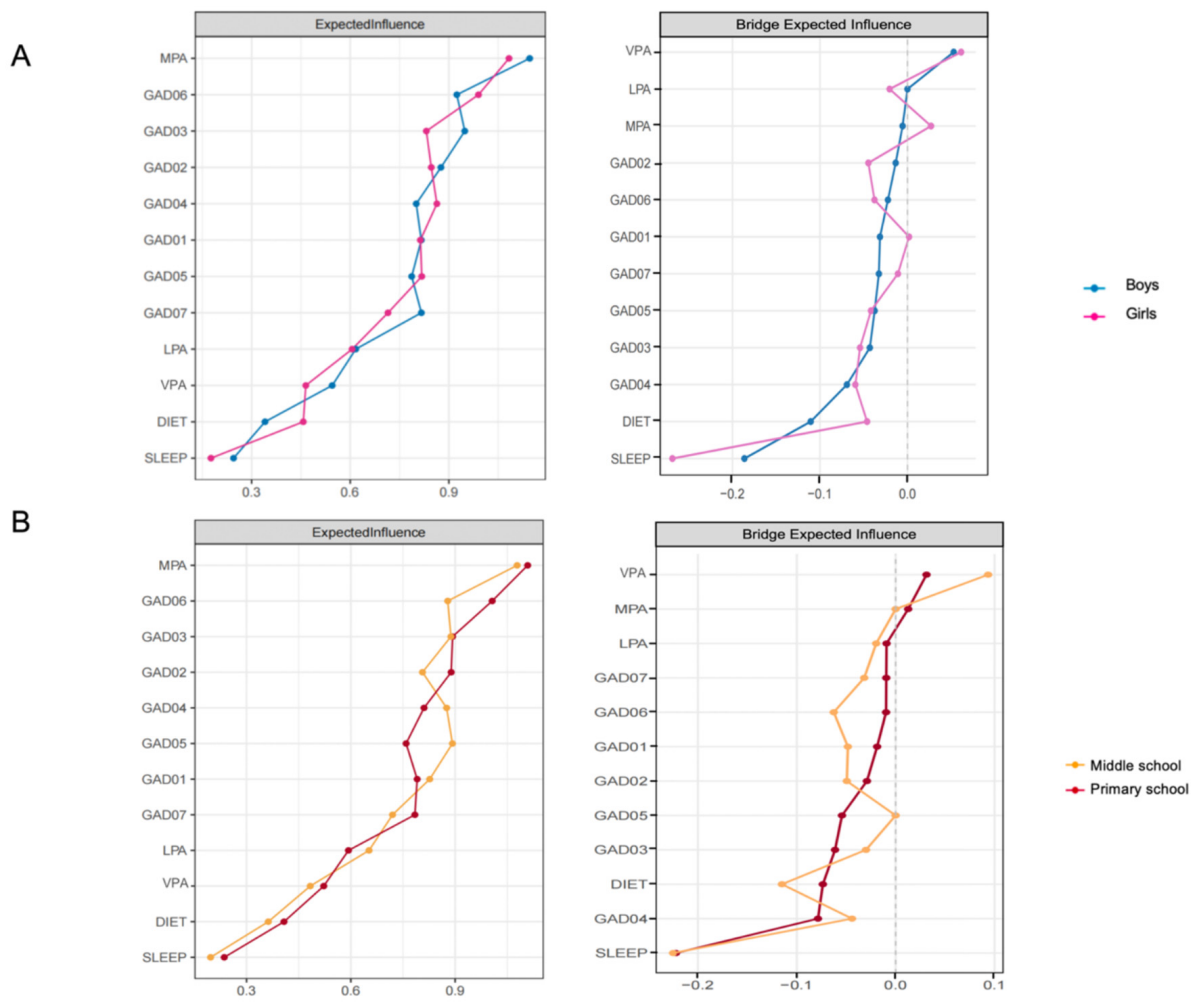
Expected influence and bridge expected influence across gender and grade groups. See Table 1 for node abbreviations. **(A)** presents gender-based comparisons of expected influence and bridge expected influence (boys vs. girls). **(B)** presents grade-based comparisons of expected influence and bridge expected influence (primary school vs. middle school).

Figure 2 presents gender-specific network structures. MPA (EI_male_ = 1.10, EI_female_ = 1.10) was the most central node in both genders, though the second most central symptom differed: GAD03 (EI = 0.95) in males vs. GAD06 (EI = 0.99) in females. Bridge EI analysis highlighted notable gender differences. In males, diet (bridge EI = −0.110) and sleep (bridge EI = −0.190) served as primary bridge nodes, with GAD04 and GAD03 demonstrating moderate cross-cluster effects. In females, sleep (bridge EI = −0.270) emerged as the dominant bridge node, indicating substantially stronger cross-cluster influence. GAD04, GAD03, and diet also showed smaller bridge contributions. Notably, physical activity variables again showed negligible bridge influence in both genders. Males displayed stronger edges for SLEEP-DIET and LPA-MPA, whereas females showed stronger symptom-symptom edges, particularly GAD06-GAD07 and GAD03-GAD04. Physical activity edges were weak across both groups (Supplementary Material 6, 7).

NCT results showed no significant differences in overall network structure (*p* = 0.263) or global expected influence (*p* = 0.358). However, five edges differed significantly by gender: VPA-MPA (*p* = 0.040), VPA-GAD01 (*p* = 0.044), GAD04-GAD06 (*p* = 0.024), DIET-GAD07 (*p* = 0.050), and GAD05-GAD07 (*p* = 0.036). Bridge centrality invariance indicated a significant gender difference only for sleep (*p* = 0.033), confirming stronger sleep-mediated bridging in females. GAD04 was the strongest anxiety-side bridge node in both genders, with larger magnitude in females (BEI = −0.054) compared with males (BEI = −0.040).

### 3.4 Network structure analysis across school stages

Primary school students showed stronger behavior-symptom connections, particularly negative edges such as SLEEP-GAD03, SLEEP-GAD04, and diet-related links (e.g., DIET-GAD04). Middle school students demonstrated more consolidated symptom-symptom connectivity, with stronger positive edges including GAD06-GAD07 and GAD03-GAD04, while behavior-related edges weakened with age (Supplementary Material 10, 11). As shown in Figure 2B, EI analysis showed that MPA remained the most central node in both groups (EI = 1.10). Central anxiety symptoms differed slightly: GAD06 and GAD03 were most central in primary school, whereas GAD03 and GAD06 were most central in middle. In bridge centrality analysis, sleep again emerged as the strongest bridge node in both grades (primary: −0.220; middle: −0.230). Diet and several anxiety symptoms (GAD06, GAD04, GAD03) also contributed moderate bridge effects. Physical activity variables exhibited minimal bridge influence across grades (Supplementary Material 12).

NCT revealed no significant differences in global network structure (*p* = 0.560) or global expected influence (*p* = 0.630), indicating stable macro-level architecture. However, four edges differed significantly by grade: VPA-MPA (*p* = 0.003), DIET-GAD01 (*p* = 0.043), VPA-GAD04 (*p* = 0.043), and DIET-GAD07 (*p* = 0.028).

Finally, stability analyses were conducted for all networks. The results showed that the CS coefficients ranged from 0.67 to 0.75 across networks, indicating that all network models reached the highest level of stability tested in this study.

## 4 Discussion

This network analysis provides a more fine-grained description of the associations among lifestyle behaviors and anxiety symptoms in children and adolescents. Moderate-intensity physical activity emerged as the most central node, and sleep showed the strongest bridging role between lifestyle behaviors and anxiety symptoms. These findings help characterize clinically relevant correlational patterns within the lifestyle-anxiety network and provide candidate associations for future longitudinal and experimental research.

The consistent centrality of MPA across all network models suggests it may play an important connecting role within the lifestyle-anxiety network, though the cross-sectional design precludes causal conclusions. This pattern is broadly consistent with prior literature linking moderate-intensity exercise to lower anxiety levels in youth (Bernard et al., 2018). Recent cohort studies demonstrate a clear dose-response relationship, showing that 30–59 min of daily MPA is associated with approximately 56% lower odds of mental health problems, with no additional benefit beyond this range (Zhang et al., 2025). The central position of MPA in our network structure is consistent with meta-analytic findings showing modest anxiety reductions following moderate-intensity exercise in adolescents (Li et al., 2023).

The superior effects of moderate relative to vigorous exercise may be rooted in distinct physiological and psychosocial pathways. Moderate activity elevates heart rate to 50–70% of maximal capacity, eliciting optimal endorphin release and mood enhancement without triggering excessive autonomic activation, which may inadvertently mimic anxiety-related arousal in vulnerable youth (Perron et al., 2011; DiGiacomo et al., 2019; Jäger et al., 2024). Our finding that vigorous-intensity physical activity showed minimal bridge influence further indicates that vigorous exercise contributes little to the integration of lifestyle and anxiety domains. Moreover, moderate activities often incorporate social elements—such as play, sports, or group exercise—which promote anxiety reduction through peer interactions and mastery experiences, mechanisms shown to be particularly beneficial for social anxiety (Chen et al., 2024; Zhang et al., 2025). From a public health perspective, the widespread inactivity among adolescents—particularly girls, over 80% of whom fail to meet vigorous activity guidelines (Guthold et al., 2020) —highlights the potential relevance of accessible moderate activities in future prevention-oriented research and practice discussions.

Our results indicate that sleep was the strongest lifestyle-related node and the most influential bridge connecting lifestyle behaviors with anxiety symptoms. A supplementary item-level sensitivity analysis further suggested that the self-developed sleep and diet nodes should be interpreted cautiously. Specifically, the constituent sleep- and diet-related items did not contribute uniformly when entered separately into the network, and this heterogeneity was particularly evident for sleep. These findings indicate that the primary-network sleep and diet nodes are better understood as pragmatic behavioral summary indicators retained for parsimony, rather than as validated unitary constructs. Accordingly, the prominent role of the sleep node in the primary analysis may reflect the influence of specific sleep-related indicators rather than a uniformly shared sleep dimension. Sleep showed strong negative partial correlations with generalized worry (GAD03) and irritability (GAD06). This pattern is consistent with prior literature suggesting that sleep disturbances are closely linked to anxiety across symptom domains rather than appearing merely as secondary correlates (Kim et al., 2022; Moon and Woo, 2025). Recent network studies similarly identify sleep as a critical bridge between mental health and lifestyle domains (Luo et al., 2025). Neurobiological research provides further grounding: sleep deprivation disrupts mPFC-amygdala connectivity, reducing top-down regulation and heightening emotional reactivity, thereby reinforcing an “anxiety-insomnia-heightened anxiety” cycle (Goldstein and Walker, 2014). Given its prominent bridge position in the primary composite-based network, sleep may represent a useful correlational signal for future intervention research; however, the supplementary item-level analysis suggests that this effect should not be interpreted as evidence for a single coherent sleep construct.

Gender-specific network patterns suggested differences in how lifestyle-related and symptom-related variables were associated across boys and girls. While the Network Comparison Test (NCT) supported global structural invariance between boys and girls, sleep demonstrated stronger bridge centrality in girls. This suggests that the statistical association between lifestyle factors and anxiety may be more strongly characterized by sleep-related bridge centrality in females. This is consistent with evidence that pubertal fluctuations in estradiol and progesterone heighten circadian vulnerability and increase risk for delayed sleep phase and insomnia among adolescent girls (Pengo et al., 2018). These findings suggest that sleep-related factors may deserve closer examination in future longitudinal research involving girls, including whether CBT-I components targeting pre-sleep rumination may be especially relevant in this group (Yang and Lei, 2025). Stronger GAD06-GAD07 associations in girls also indicate tighter coupling between irritability and fear symptoms, aligning with research showing greater emotional reactivity and cross-symptom amplification among adolescent females (Zahn-Waxler et al., 2008; Oldehinkel and Bouma, 2011). Heightened amygdala responsivity (Guyer et al., 2009) and reduced prefrontal regulation efficiency (Ordaz et al., 2013) provide neurobiological explanations for this rapid symptom spread. In contrast, boys displayed weaker symptom connectivity but stronger clustering among lifestyle behaviors, suggesting that they may benefit more from behavioral activation approaches emphasizing consistent routines across sleep, diet, and activity.

Developmental differences across school stages also offer important insight. The stronger negative connections between sleep and diet with anxiety in primary school students indicate that anxiety in younger children may be more closely associated with daily behavioral patterns. This aligns with evidence that childhood anxiety often manifests through physiological and behavioral channels, including irritability, restlessness, and fatigue (Coxon and Gibson, 2025). In contrast, middle school students showed stronger symptom-symptom connectivity (e.g., GAD03-GAD04, GAD06-GAD07), suggesting that with increasing age and cognitive maturity, anxiety symptoms become more mutually reinforcing, a pattern consistent with research showing heightened susceptibility to persistent worry during adolescence (Liu et al., 2024). Although macro-level network organization remained stable across age groups, several edge-level differences (e.g., VPA-MPA, DIET-GAD07) suggest developmental shifts in the specific pathways linking behaviors and anxiety. These patterns may have implications for future intervention research: behavioral patterns related to sleep and diet may warrant closer attention in younger children, although the composite nature of these nodes and the cross-sectional design limit intervention-oriented interpretation (Bacaro et al., 2024), whereas the stronger symptom-symptom connectivity observed in adolescents may indicate the value of further examining approaches that target reinforcing anxiety loops, such as cognitive-behavioral strategies. The repeated prominence of sleep as a bridge node across both age groups suggests that sleep-related factors may be an important feature to examine further in longitudinal and experimental studies across childhood and adolescence (Tao et al., 2024).

## 5 Limitations and future directions

First, the cross-sectional design precludes causal inference; central or bridge nodes should be interpreted as correlational signals rather than causal leverage points. These findings are hypothesis-generating, and claims about mechanisms or intervention relevance require longitudinal or experimental evidence. Second, the reliance on self-reported measures may introduce biases. The IPAQ-SF may not be appropriate for the youngest participants, and measurement invariance of the GAD-7 across school stages was not formally tested. Sleep and dietary behaviors were assessed using brief self-developed items. Supplementary item-level analyses suggested that the central role of MPA was preserved, whereas the constituent sleep- and diet-related items showed heterogeneous contributions, particularly for sleep. Future research should employ validated instruments such as the Pittsburgh Sleep Quality Index. Third, estimated edges were conditional only on variables included in the network. Although a stage-adjusted sensitivity analysis suggested that the overall cross-domain pattern was broadly preserved, robustness to other demographic or contextual factors could not be fully assessed, limiting the ability to rule out alternative explanations. Finally, the sample was drawn from a single prefecture-level city in southwestern China. The specific city name is not disclosed to protect the privacy of minor participants. This geographic scope limits generalizability, and unequal group sizes may have reduced power for school-stage comparisons. Replication in diverse samples using validated instruments and longitudinal designs is needed before drawing conclusions about causal mechanisms or intervention relevance. In addition, unequal subgroup sizes may have reduced statistical power for detecting small edge-level differences in the network comparison test. Findings from subgroup comparisons should be replicated in larger, more balanced samples.

## 6 Conclusion

This study showed that moderate-intensity physical activity occupied the most central lifestyle-related position within the lifestyle-anxiety network, whereas sleep was the strongest bridge-related correlate linking lifestyle behaviors and anxiety symptoms. The network structure also differed across gender and developmental groups. In girls, sleep showed stronger cross-domain bridge connectivity, whereas in younger students, behavior-symptom associations appeared more prominent. At the same time, supplementary analyses indicated that the sleep and diet nodes should be interpreted cautiously, particularly because the constituent sleep-related indicators did not contribute uniformly at the item level. In addition, stage-adjusted analyses suggested that the overall cross-domain pattern was preserved, although some rank-order changes were observed. Overall, these findings provide a fine-grained description of how lifestyle-related and anxiety-related variables co-occur in children and adolescents. Rather than identifying definitive intervention targets, the present results identify correlational patterns and group-specific features that warrant further investigation in future longitudinal and experimental research on youth anxiety and health-related behaviors.

## Data Availability

The data supporting the findings of this study are available from the corresponding author upon reasonable request.
