# Supplementary material for: Network analysis of lifestyle behaviors and anxiety in children and adolescents: gender and school-stage heterogeneity
Source: Front Psychol. 2026 May 18;17:1803654. doi: 10.3389/fpsyg.2026.1803654 (PMC13224473; doi:10.3389/fpsyg.2026.1803654)
Supplement: Supplementary file 1 [file Data_Sheet_1.pdf]

**Supplemental materials**

1. Adjacency matrix of the lifestyle behaviours and anxiety symptoms network
2. Edge weights of the lifestyle behaviours and anxiety symptoms network
3. Bootstrapped pair-wise comparsion of bridges in lifestyle behaviours and anxiety symptoms network
4. Expected influence centrality and bridge expected influence in the lifestyle behaviours and anxiety symptoms network
5. Adjacency matrices of the lifestyle behaviours and anxiety symptoms network by gender (boys and girls)
6. Edge weights of the lifestyle behaviours and anxiety symptoms network by gender (boys and girls)
7. Bootstrapped pairwise comparison of bridge expected influence in the lifestyle behaviours and anxiety symptoms network by gender (boys and girls)
8. Expected influence and bridge expected influence centrality of the lifestyle behaviours and anxiety symptoms network by gender (boys and girls)
9. Adjacency matrices of the lifestyle behaviours and anxiety symptoms network by grade level (primary and middle school)
10. Edge weights of the lifestyle behaviours and anxiety symptoms network by grade level (primary and middle school)
11. Bootstrapped pairwise comparison of bridge expected influence in the lifestyle behaviours and anxiety symptoms network by grade level (primary and middle school)
12. Expected influence and bridge expected influence centrality of the lifestyle behaviours and anxiety symptoms network by grade level (primary and middle school)
13. Correlation stability coefficient of the network
14. Centrality and Bridge Centrality Indices in the Sensitivity Analysis Excluding Grades 1-2
15. Grade-Specific Item-Level Response Distributions for the GAD-7
16. The corresponding item-level centrality and bridge results
17. Item wording and scoring of the self-developed sleep and diet measures
18. Node predictability ($R^2$) of the lifestyle behaviours and anxiety symptoms network

Supplemental material 1

Adjacency matrix of the lifestyle behaviours and anxiety symptoms network

| | VPA | MPA | LPA | SLEEP | DIET | GAD01 | GAD02 | GAD03 | GAD04 | GAD05 | GAD06 | GAD07 |
|---|---|---|---|---|---|---|---|---|---|---|---|---|
| VPA | 0 | 0.45 | 0 | 0 | 0 | 0 | 0.01 | 0 | 0.02 | 0.01 | 0.00 | 0.02 |
| MPA | 0.45 | 0 | 0.59 | 0 | 0.07 | 0 | 0 | 0 | 0 | 0 | 0.01 | 0 |
| LPA | 0 | 0.59 | 0 | 0.04 | 0 | -0.01 | 0 | 0 | 0 | 0 | 0 | -0.01 |
| SLEEP | 0 | 0 | 0.04 | 0 | 0.41 | -0.02 | -0.04 | -0.06 | -0.04 | -0.01 | -0.05 | -0.02 |
| DIET | 0 | 0.07 | 0 | 0.41 | 0 | 0 | 0 | 0 | -0.05 | -0.03 | 0 | 0 |
| GAD01 | 0 | 0 | -0.01 | -0.02 | 0 | 0 | 0.32 | 0.17 | 0.13 | 0.06 | 0.13 | 0.03 |
| GAD02 | 0.01 | 0 | 0 | -0.04 | 0 | 0.32 | 0 | 0.20 | 0.10 | 0.14 | 0.04 | 0.09 |
| GAD03 | 0 | 0 | 0 | -0.06 | 0 | 0.17 | 0.20 | 0 | 0.20 | 0.04 | 0.19 | 0.15 |
| GAD04 | 0.02 | 0 | 0 | -0.04 | -0.05 | 0.13 | 0.10 | 0.20 | 0 | 0.17 | 0.16 | 0.13 |
| GAD05 | 0.01 | 0 | 0 | -0.01 | -0.03 | 0.06 | 0.14 | 0.04 | 0.17 | 0 | 0.26 | 0.18 |
| GAD06 | 0.00 | 0.01 | 0 | -0.05 | 0 | 0.13 | 0.04 | 0.19 | 0.16 | 0.26 | 0 | 0.21 |
| GAD07 | 0.02 | 0 | -0.01 | -0.02 | 0 | 0.03 | 0.09 | 0.15 | 0.13 | 0.18 | 0.21 | 0 |

Supplemental material 2
Edge weights of the lifestyle behaviours and anxiety symptoms network

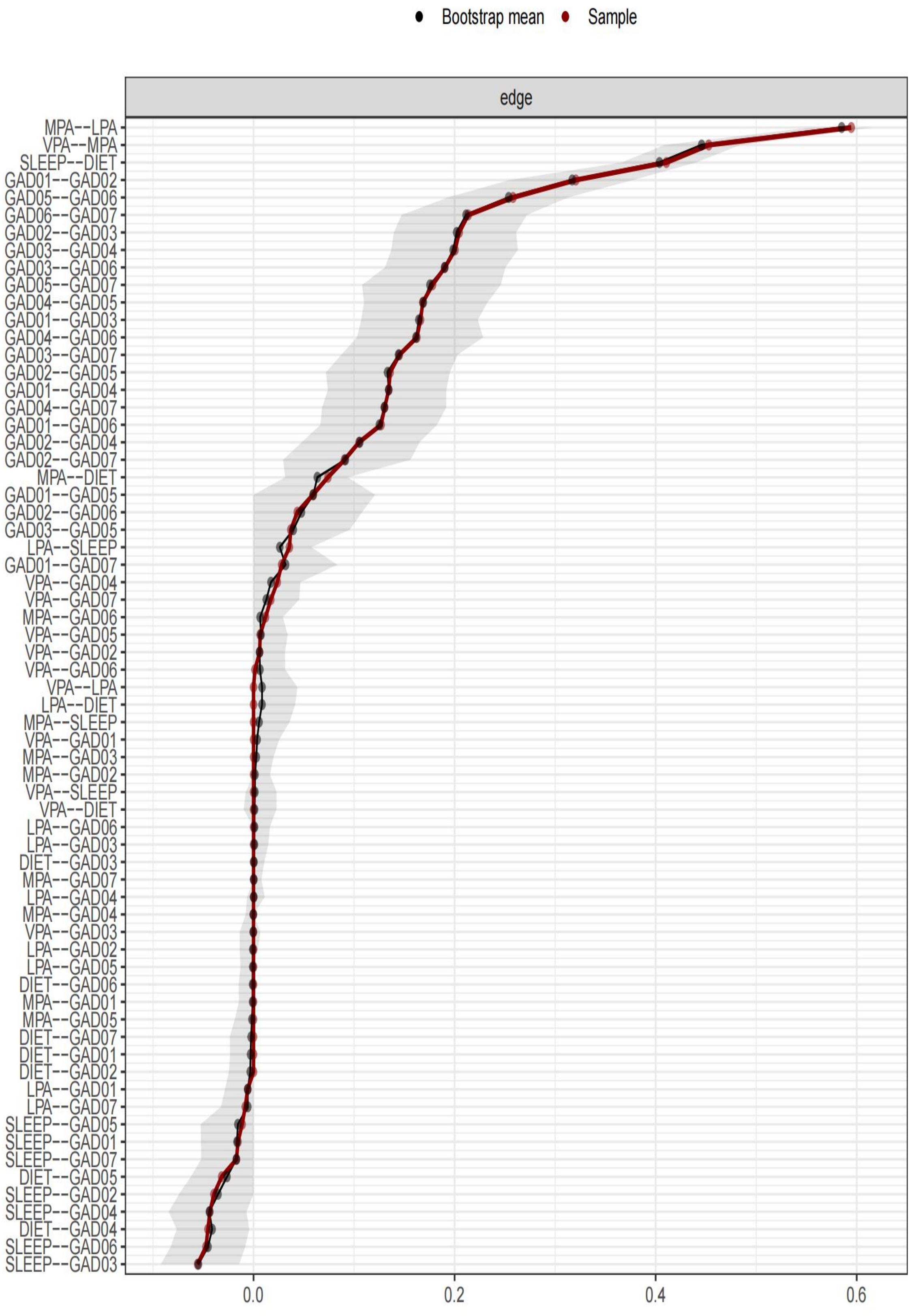

Supplemental material 3

Bootstrapped pair-wise comparsion of bridges in lifestyle behaviours and anxiety symptoms network

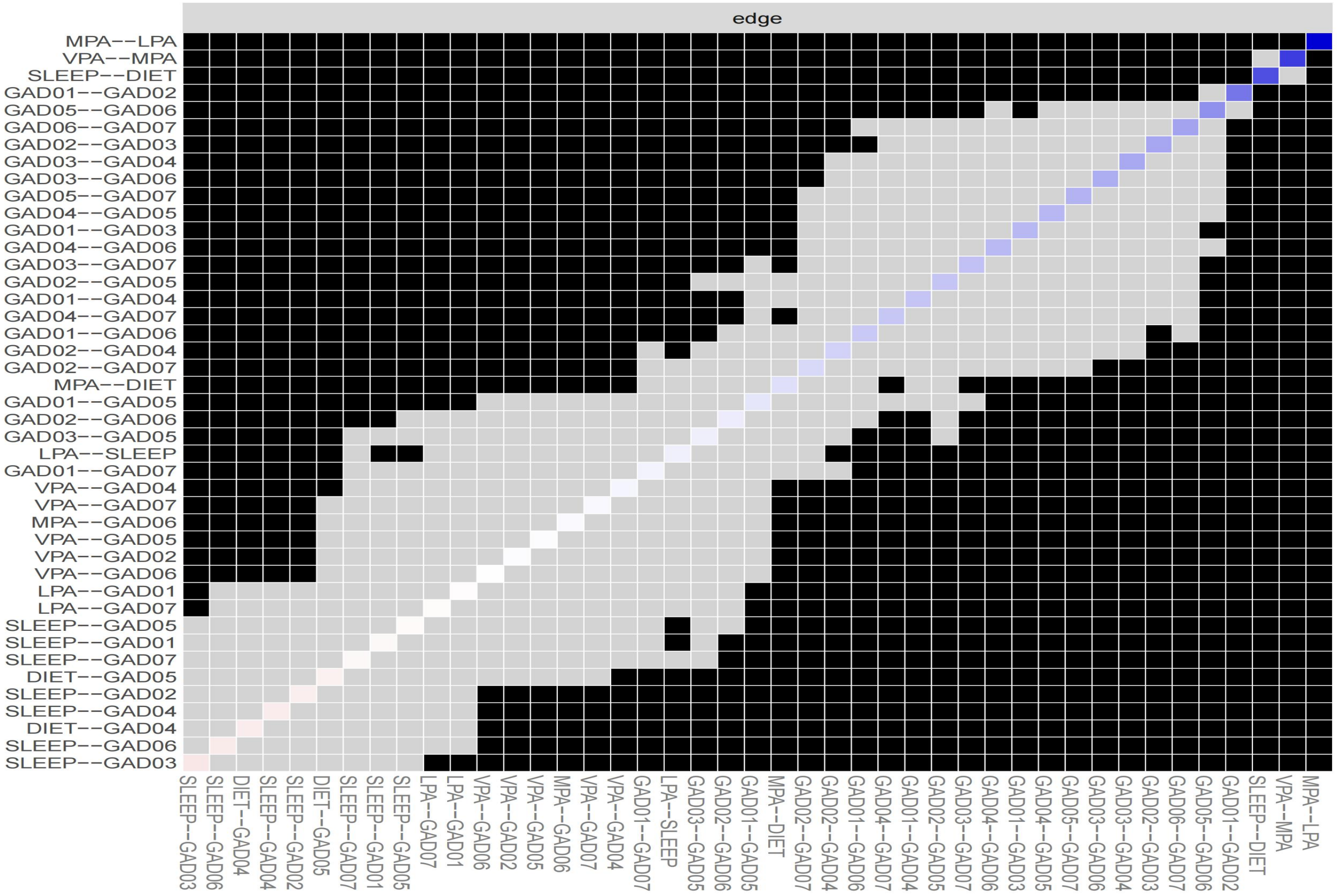

Supplemental material 4

Expected influence centrality and bridge expected influence in the lifestyle behaviours and anxiety symptoms network

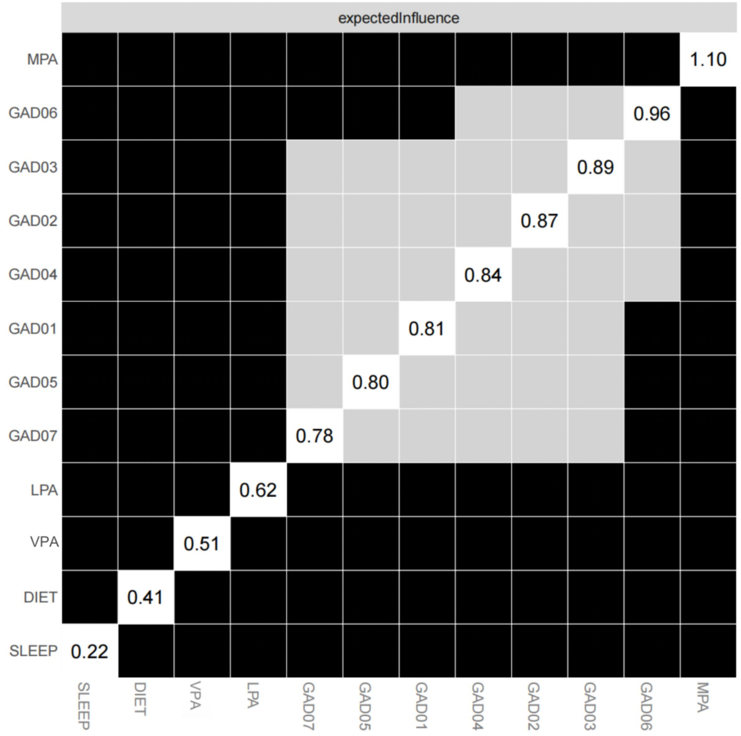


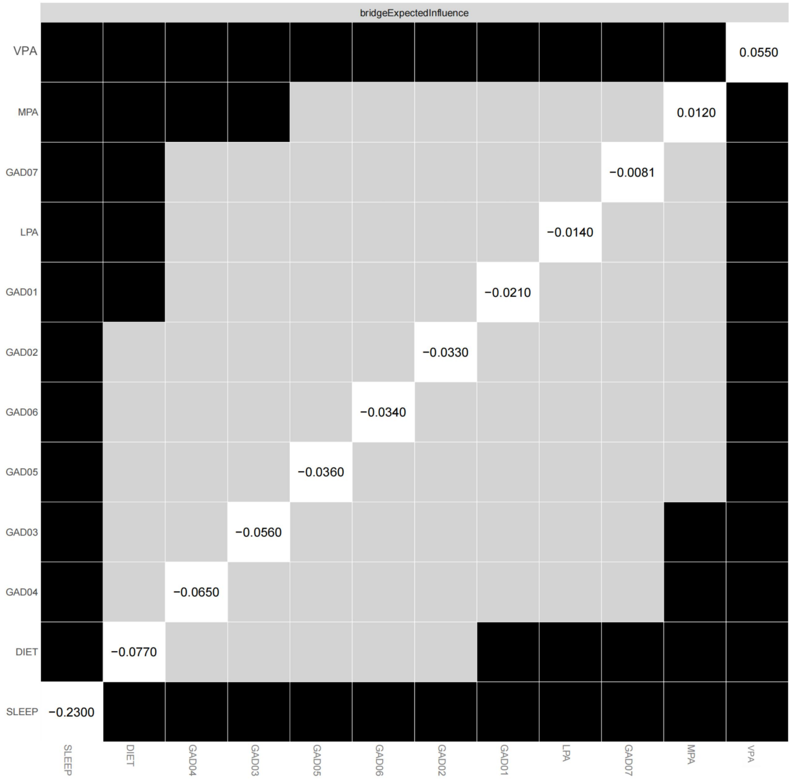

Supplemental material 5

Adjacency matrices of the lifestyle behaviours and anxiety symptoms network by gender (boys and girls)

| | VPA | MPA | LPA | SLEEP | DIET | GAD01 | GAD02 | GAD03 | GAD04 | GAD05 | GAD06 | GAD07 |
|---|---|---|---|---|---|---|---|---|---|---|---|---|
| VPA | 0 | 0.40 | 0 | 0 | 0 | 0.03 | 0 | 0 | 0.02 | 0 | 0 | 0.02 |
| MPA | 0.48 | 0 | 0.59 | 0 | 0.06 | 0 | 0 | 0.01 | 0 | 0 | 0.01 | 0 |
| LPA | 0.01 | 0.58 | 0 | 0.02 | 0.02 | -0.01 | 0 | 0 | 0 | 0 | 0 | -0.01 |
| SLEEP | 0 | 0.02 | 0.03 | 0 | 0.43 | 0 | -0.04 | -0.07 | -0.05 | -0.04 | -0.05 | -0.02 |
| DIET | 0 | 0.07 | 0 | 0.38 | 0 | -0.02 | 0 | 0 | -0.03 | 0 | 0 | 0 |
| GAD01 | 0 | -0.01 | 0 | -0.03 | 0 | 0 | 0.30 | 0.14 | 0.12 | 0.06 | 0.16 | 0.04 |
| GAD02 | 0.02 | 0 | 0 | -0.03 | 0 | 0.33 | 0 | 0.20 | 0.15 | 0.13 | 0.06 | 0.05 |
| GAD03 | 0 | 0 | 0 | -0.04 | 0 | 0.20 | 0.20 | 0 | 0.20 | 0.09 | 0.17 | 0.09 |
| GAD04 | 0.02 | 0 | 0 | -0.04 | -0.05 | 0.15 | 0.06 | 0.20 | 0 | 0.12 | 0.23 | 0.11 |
| GAD05 | 0.01 | 0 | 0 | 0 | -0.04 | 0.07 | 0.14 | 0 | 0.21 | 0 | 0.21 | 0.24 |
| GAD06 | 0.01 | 0 | 0 | -0.04 | 0 | 0.09 | 0.03 | 0.21 | 0.08 | 0.30 | 0 | 0.20 |
| GAD07 | 0 | 0 | 0 | -0.01 | -0.02 | 0.02 | 0.13 | 0.19 | 0.17 | 0.10 | 0.24 | 0 |

Note. See Table 1 for node abbreviations. The upper triangle of the matrix represents the girls’ network, and the lower triangle represents the boys’ network.

Supplemental material 6

Edge weights of the lifestyle behaviours and anxiety symptoms network by gender (boys and girls)

A

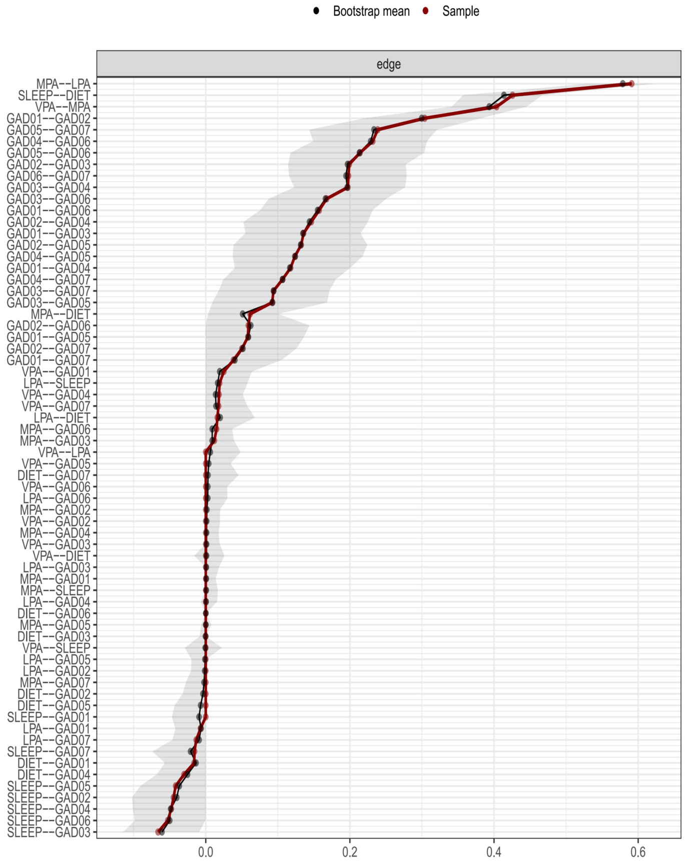


B

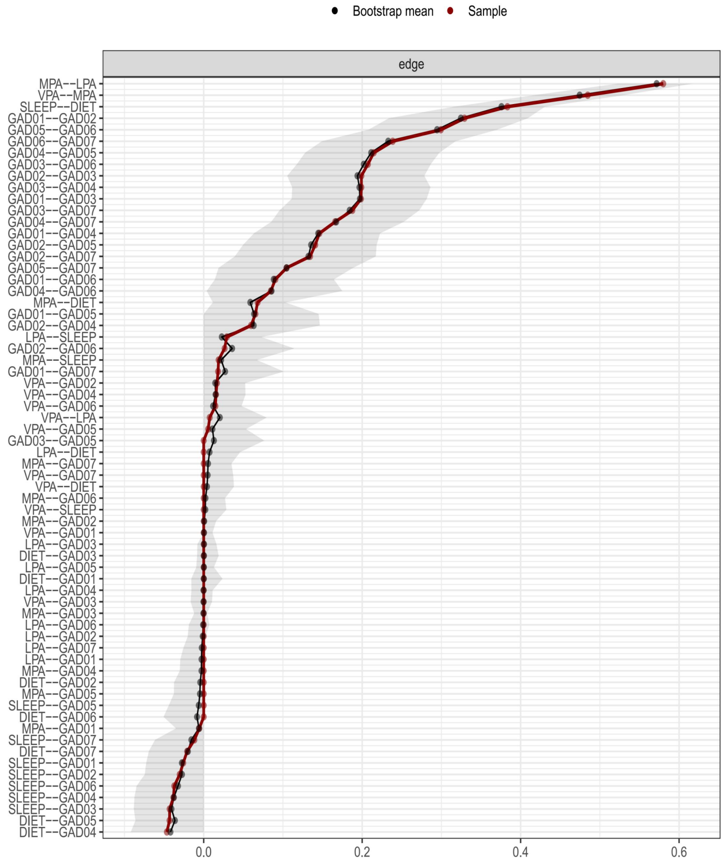


Note. See Table 1 for node abbreviations. A is the girls' network, and B is the boys' network.

Supplemental material 7

Bootstrapped pairwise comparison of bridge expected influence in the lifestyle behaviours and anxiety symptoms network by gender (boys and girls)

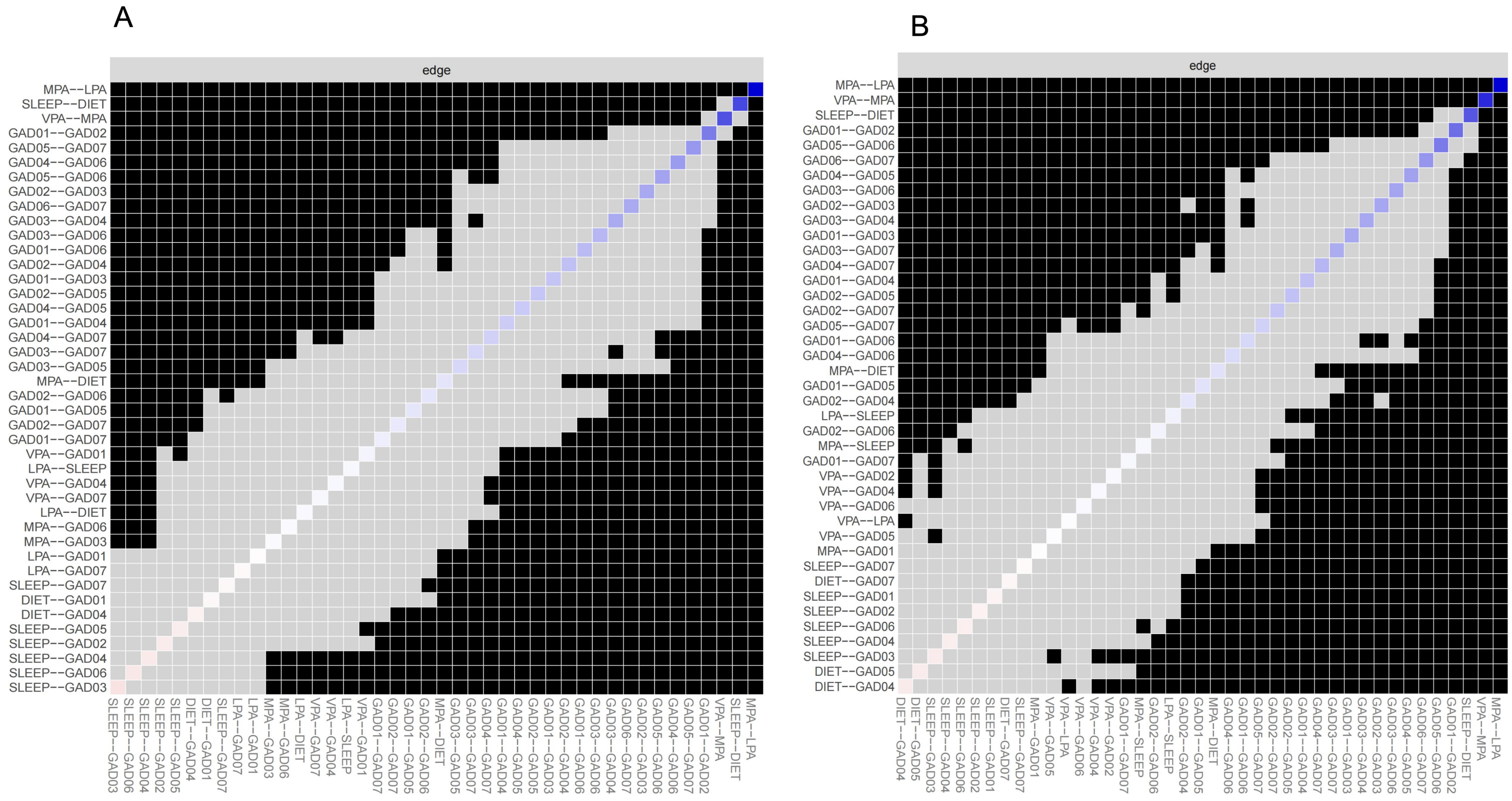


Note. See Table 1 for node abbreviations. A is the girls' network, and B is the boys' network.

Supplemental material 8

Expected influence and bridge expected influence centrality of the lifestyle behaviours and anxiety symptoms network by gender (boys and girls)

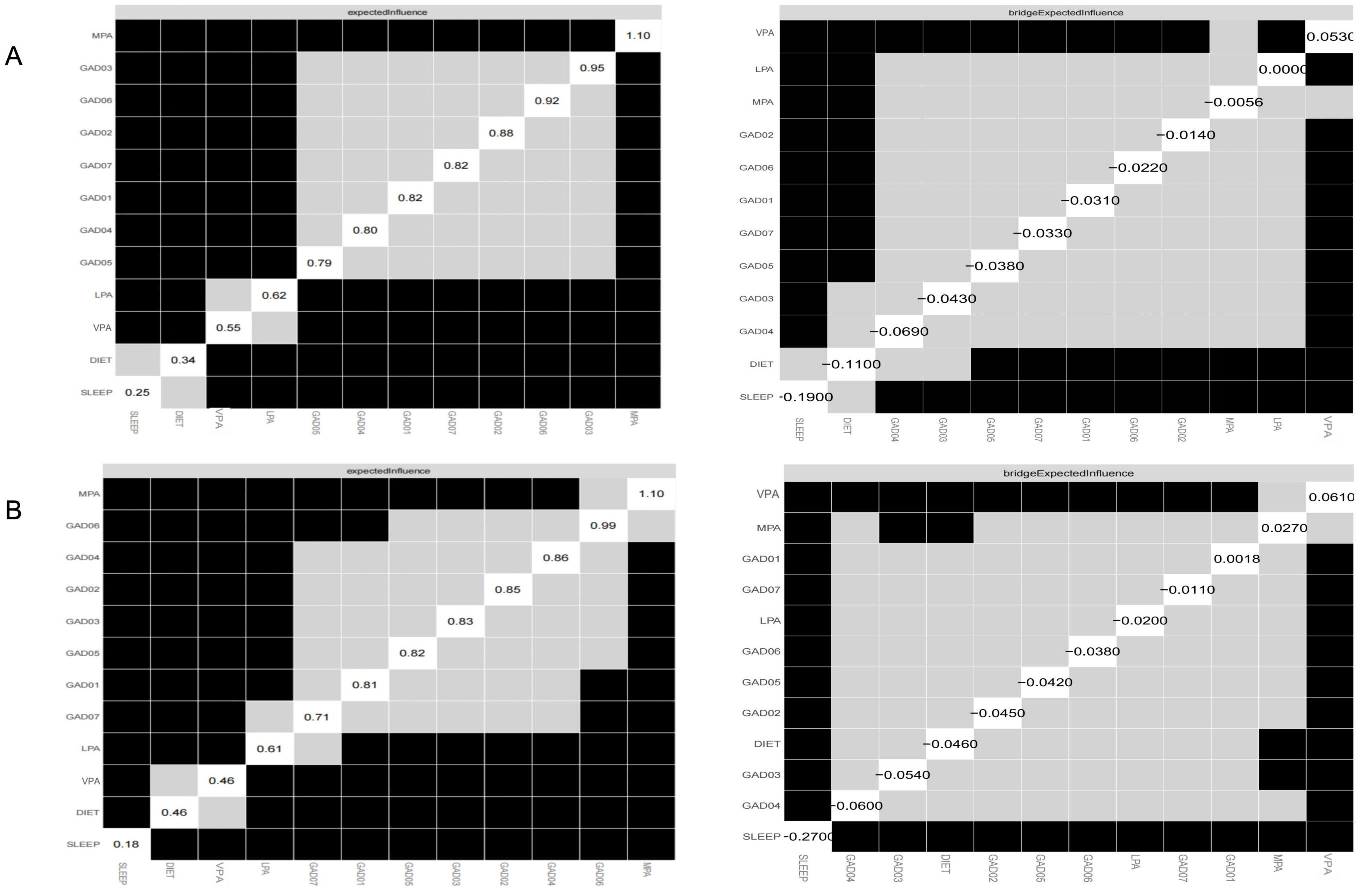


Note. See Table 1 for node abbreviations. A is the girls' network, and B is the boys' network.

Supplemental material 9

Adjacency matrices of the lifestyle behaviours and anxiety symptoms network by grade level (primary and middle school)

| | VPA | MPA | LPA | SLEEP | DIET | GAD01 | GAD02 | GAD03 | GAD04 | GAD05 | GAD06 | GAD07 |
|---|---|---|---|---|---|---|---|---|---|---|---|---|
| VPA | 0 | 0.36 | 0.01 | 0 | 0.01 | 0 | 0.01 | 0 | 0.05 | 0 | 0 | 0.04 |
| MPA | 0.49 | 0 | 0.62 | 0.01 | 0.09 | 0 | 0 | 0 | 0 | 0 | 0 | 0 |
| LPA | 0 | 0.56 | 0 | 0.04 | 0 | -0.02 | 0 | 0 | 0 | 0 | 0 | 0 |
| SLEEP | 0 | 0 | 0.04 | 0 | 0.38 | 0 | -0.06 | -0.03 | -0.04 | 0 | -0.06 | -0.04 |
| DIET | 0 | 0.05 | 0.01 | 0.42 | 0 | -0.03 | 0 | 0 | -0.05 | 0 | 0 | -0.03 |
| GAD01 | 0 | 0 | 0 | -0.02 | 0 | 0 | 0.28 | 0.16 | 0.17 | 0.08 | 0.16 | 0.04 |
| GAD02 | 0 | 0 | 0 | -0.03 | 0 | 0.34 | 0 | 0.23 | 0.06 | 0.16 | 0.05 | 0.07 |
| GAD03 | 0 | 0.01 | 0 | -0.07 | 0 | 0.17 | 0.19 | 0 | 0.17 | 0.06 | 0.16 | 0.14 |
| GAD04 | 0 | 0 | 0 | -0.05 | -0.03 | 0.11 | 0.13 | 0.22 | 0 | 0.19 | 0.21 | 0.12 |
| GAD05 | 0.01 | 0 | 0 | -0.02 | -0.04 | 0.05 | 0.12 | 0.02 | 0.17 | 0 | 0.19 | 0.21 |
| GAD06 | 0.02 | 0.01 | 0 | -0.04 | 0 | 0.11 | 0.05 | 0.21 | 0.13 | 0.29 | 0 | 0.17 |
| GAD07 | 0 | 0 | -0.01 | 0.00 | 0 | 0.03 | 0.10 | 0.15 | 0.13 | 0.16 | 0.23 | 0 |

Note. See Table 1 for node abbreviations. The upper triangle corresponds to middle school , and the lower triangle to primary school.

Supplemental material 10

Edge weights of the lifestyle behaviours and anxiety symptoms network by grade level (primary and middle school)

A

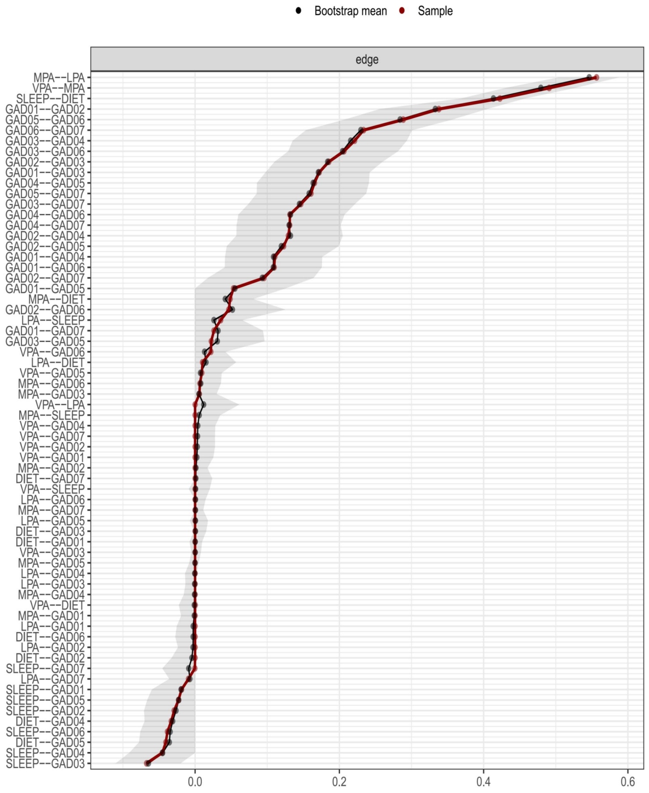


B

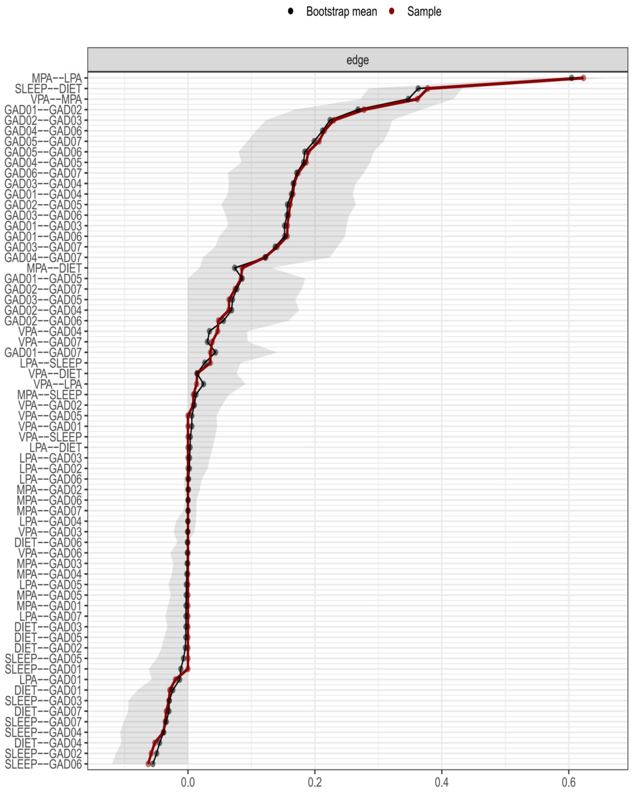


Note. See Table 1 for node abbreviations. A is the primary school network, and B is the middle school network.

Supplemental material 11

Bootstrapped pairwise comparison of bridge expected influence in the lifestyle behaviours and anxiety symptoms network by grade level (primary and middle school)

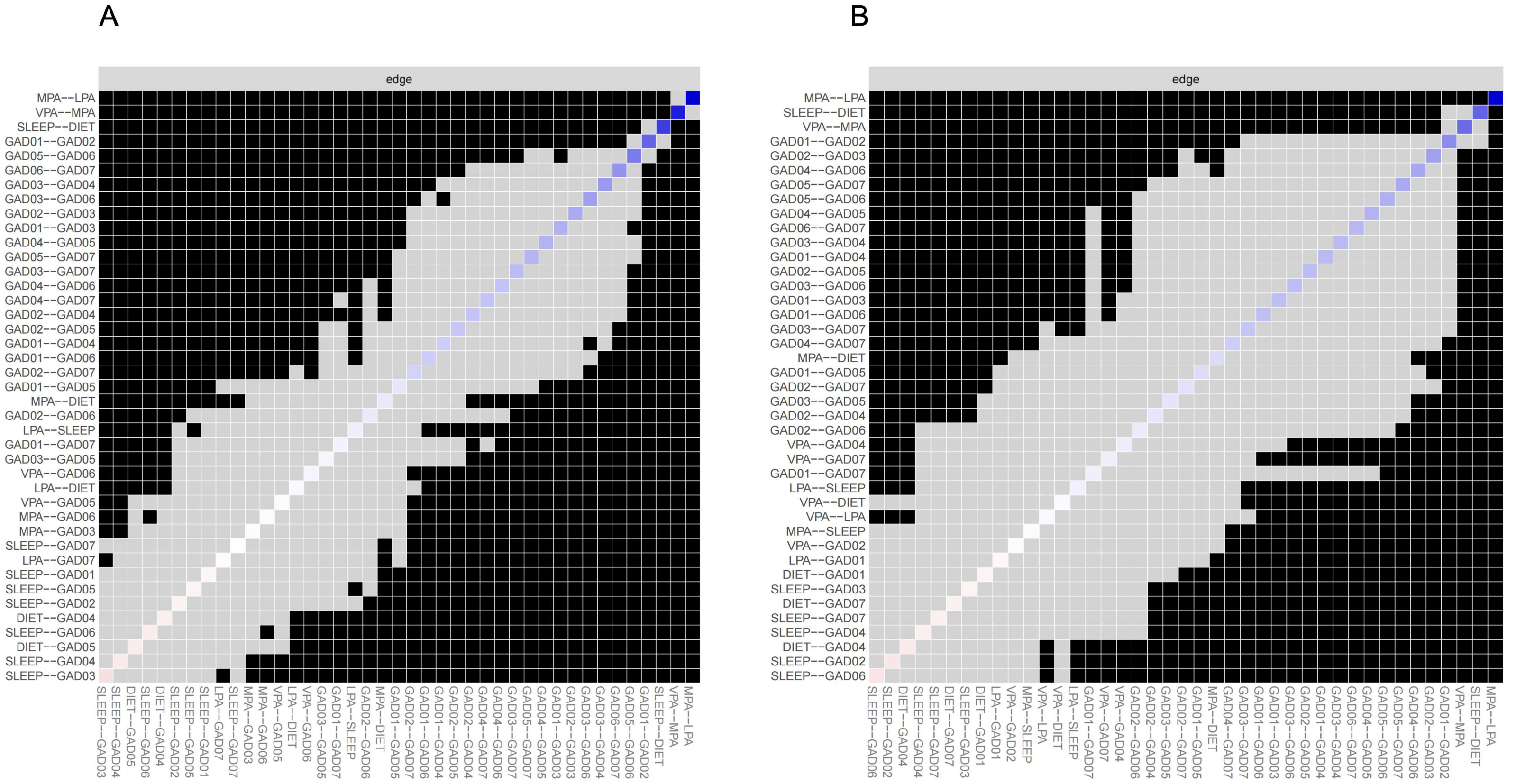

Note. See Table 1 for node abbreviations. A is the primary school network, and B is the middle school network.

Supplemental material 12

Expected influence and bridge expected influence centrality of the lifestyle behaviours and anxiety symptoms network by grade level (primary and middle school)

A

B

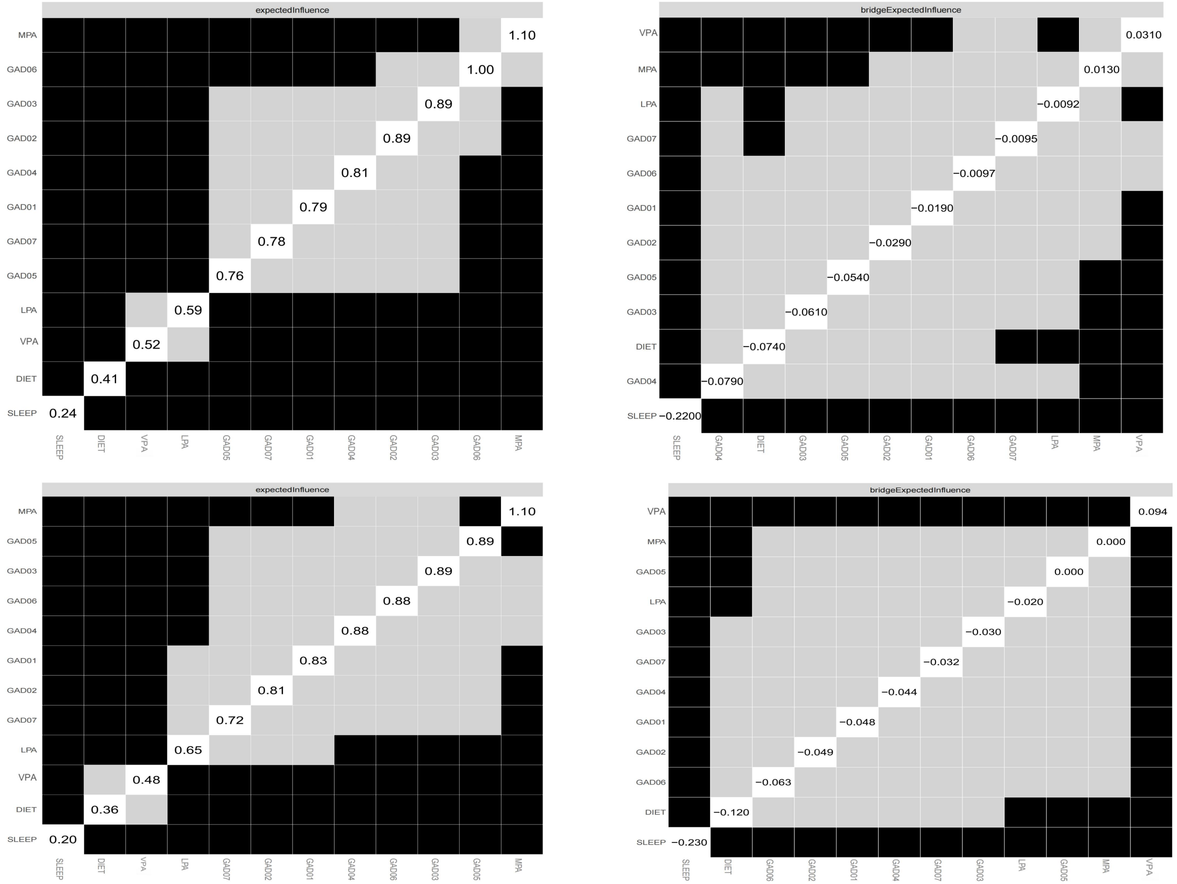


Note. See Table 1 for node abbreviations. A is the primary school network, and B is the middle school network.

Supplemental material 13

Correlation stability coefficient of the network

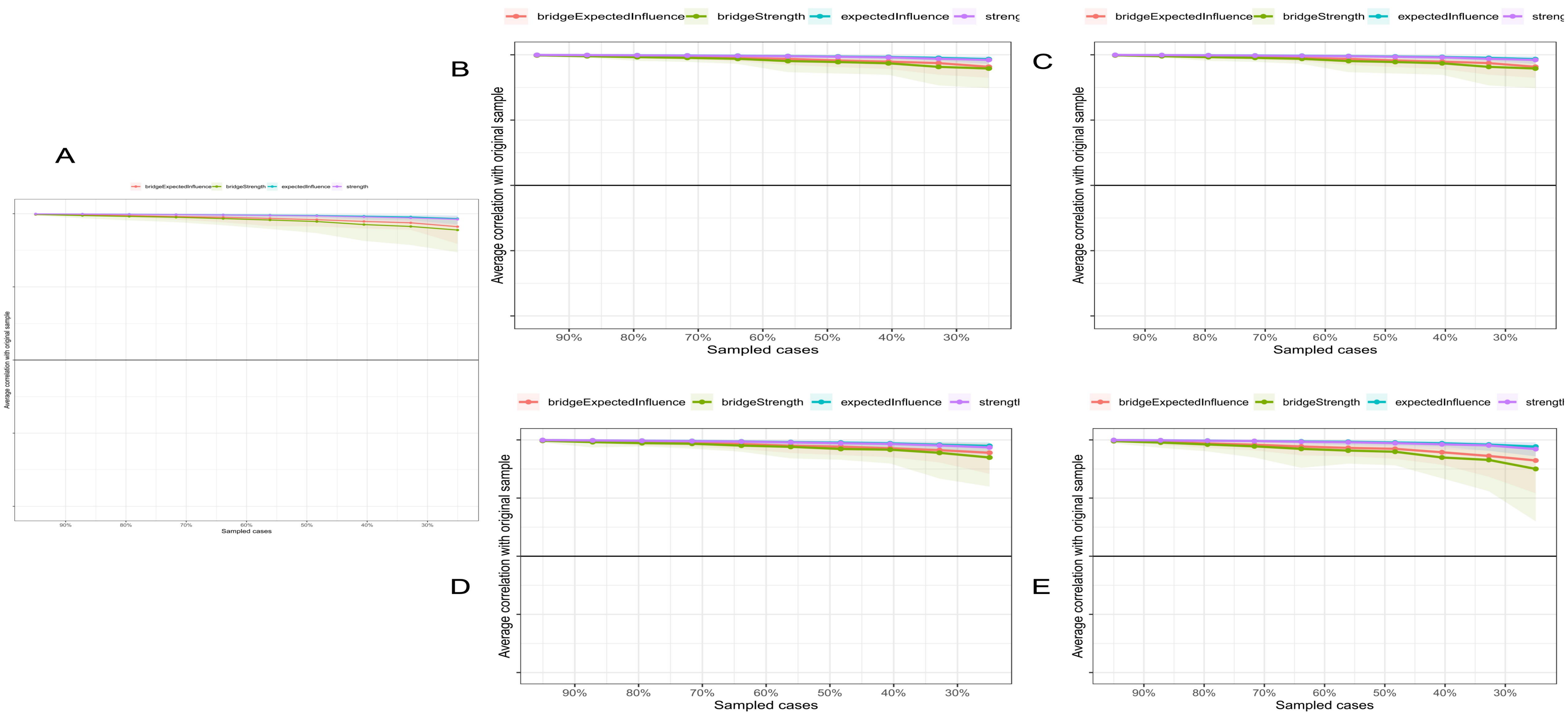


Note. See Table 1 for node abbreviations. Panel A displays the overall network. Panel B displays the primary school network, Panel C displays the junior high school network, Panel D displays the girls’ network, and Panel E displays the boys’ network.

Supplemental material 14

Centrality and Bridge Centrality Indices in the Sensitivity Analysis Excluding Grades 1–2

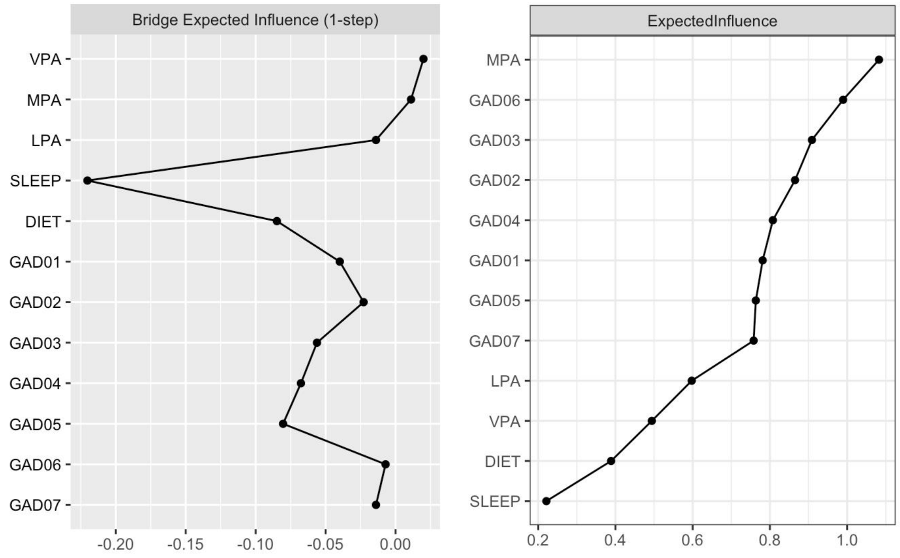

Supplemental material 15

Grade-Specific Item-Level Response Distributions for the GAD-7

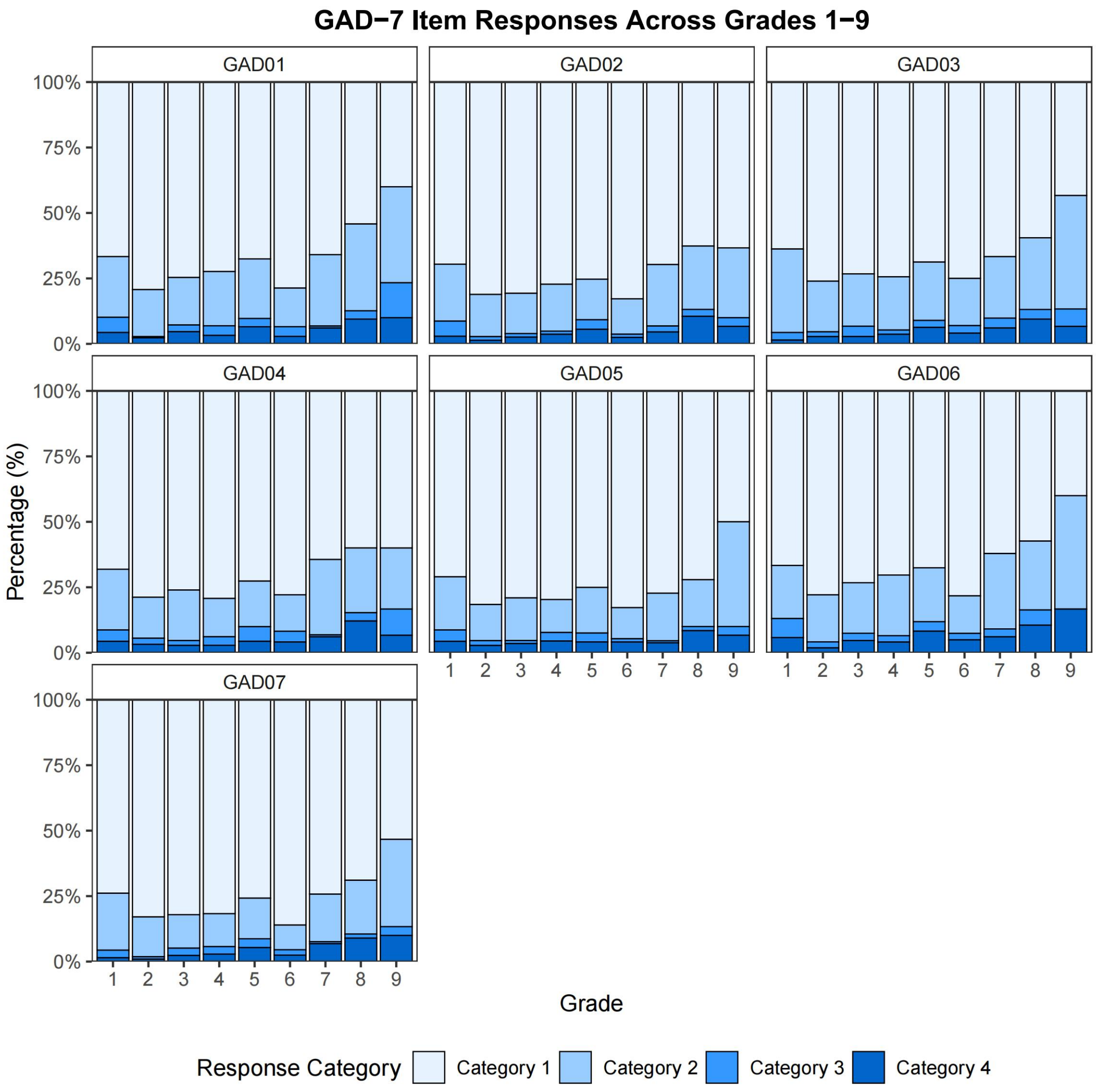

Supplemental material 16

The corresponding item-level centrality and bridge results

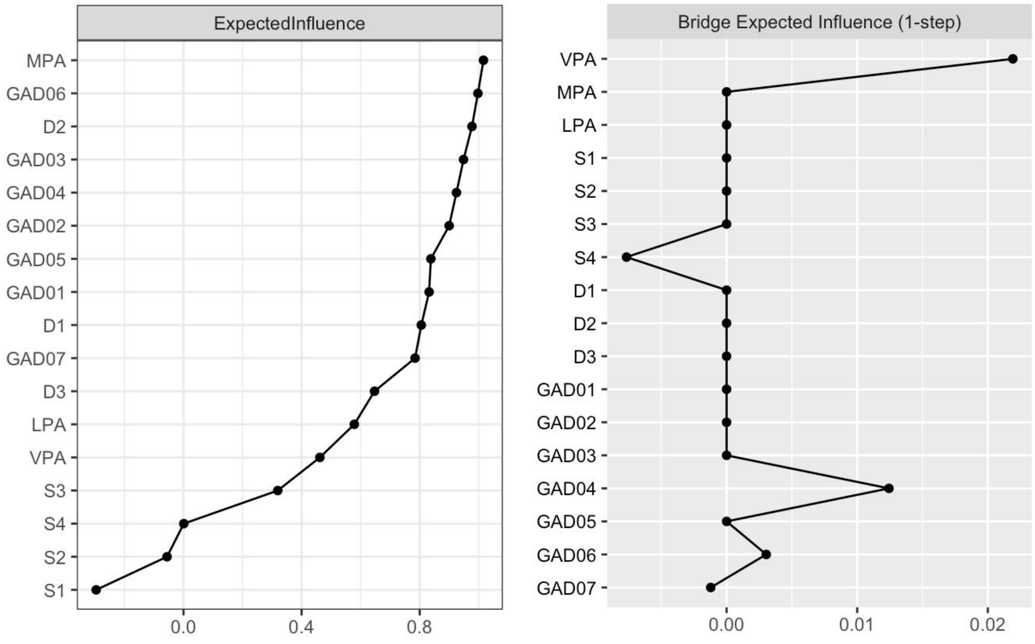

Supplemental material 17

Item wording and scoring of the self-developed sleep and diet measures

| Domain | Item | Response options | Scoring |
|---|---|---|---|
| Sleep | Daily sleep duration including naps | ≥8 hours; <8 hours | ≥8 hours = 1; <8 hours = 0 |
| | Sleep environment: sleeping with lights on | No; Yes | No = 1; Yes = 0 |
| | Regularity of sleep schedule | 1 = never, 2 = occasionally, 3 = often, 4 = always | 1–4 |
| | Frequency of insomnia or poor sleep quality | 1 = always, 2 = often, 3 = occasionally, 4 = never | 1–4 |
| Diet | Having three meals at regular times each day | 1 = never, 2 = occasionally, 3 = often, 4 = always | 1–4 |
| | Paying attention to nutritional balance in the diet | 1 = never, 2 = occasionally, 3 = often, 4 = always | 1–4 |
| | Eating vegetables and fruits | 1 = never, 2 = occasionally, 3 = often, 4 = always | 1–4 |

Supplemental material 18

Node predictability ($R^2$) of the lifestyle behaviours and anxiety symptoms network

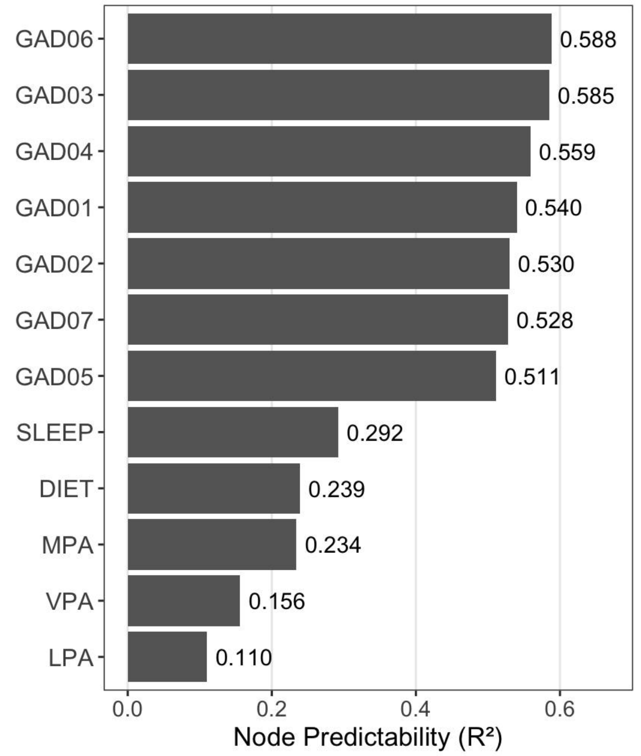


Note. See Table 1 for node abbreviations.
